# Objective sleep characteristics and longitudinal change in financial and health literacy in older adults

**DOI:** 10.1002/alz.71948

**Published:** 2026-11-01

**Authors:** Lianlian Du, Lei Yu, Tianhao Wang, Aron S. Buchman, Andrew S. P. Lim, David A. Bennett, Patricia A. Boyle

**Affiliations:** ^1^ Rush Alzheimer's Disease Center Rush University Medical Center Chicago Illinois USA; ^2^ Department of Neurological Sciences Rush University Medical Center Chicago Illinois USA; ^3^ Division of Neurology, Department of Medicine, Sunnybrook Health Sciences Centre University of Toronto Toronto Ontario Canada; ^4^ Department of Psychiatry and Behavioral Sciences Rush University Medical Center Chicago Illinois USA

**Keywords:** actigraphy, cognitive aging, daytime napping, functional aging, literacy, sleep

## Abstract

**INTRODUCTION:**

Sleep disturbances are common in older adults and linked to cognitive decline, but their association with functional abilities such as financial and health literacy is less understood.

**METHODS:**

Participants without dementia from the Rush Memory and Aging Project (*N* = 765) completed annual literacy assessments. Baseline sleep measures included self‐reported nighttime sleep duration, actigraphy‐derived nighttime sleep duration, and actigraphy‐derived daytime napping. Linear mixed‐effects models examined associations with literacy change.

**RESULTS:**

Literacy declined by ≈ 1 percentage point per year. Longer nighttime sleep was associated with higher baseline literacy and with slower decline in single‐predictor models, but the decline association was attenuated and no longer statistically significant after adjustment for daytime napping. Greater daytime napping was associated with slower decline but not baseline literacy. Self‐reported sleep was not associated with literacy trajectories, although short sleep (< 6 hours) was linked to lower baseline literacy.

**DISCUSSION:**

Objective sleep characteristics were associated with financial and health literacy trajectories in older adults, suggesting that sleep behavior may be related to changes in financial and health decision‐making abilities.

## Highlights

- Longer objective nighttime sleep was associated with higher literacy and, in single‐predictor models, slower decline; the decline association attenuated after adjustment for napping.
- Greater daytime napping was associated with slower literacy decline, with no consistent association with baseline literacy.
- Self‐reported sleep duration was not associated with literacy trajectories in continuous analyses, although short sleep (< 6 hours) was associated with lower baseline literacy.
- Findings were robust across multiple sensitivity analyses, including exclusion of participants who developed dementia.
- Sleep behavior may be associated with functional abilities relevant to real‐world decision making in later life.

## 1 BACKGROUND

Financial and health literacy refers to the ability to acquire, understand, and apply financial and health knowledge to guide everyday decisions.^1^, ^2^ These abilities are essential throughout adulthood, but they become especially consequential in later life when individuals must navigate complex decisions related to retirement distributions, long‐term financial planning, medical care, insurance coverage, and medications. A substantial literature shows that low literacy in older adults is associated with a wide range of adverse outcomes, including poorer health status, financial vulnerability, and increased mortality.^3^, ^4^, ^5^, ^6^ Thus, financial and health literacy represents a critical functional ability that supports independence and well‐being in aging populations.

Studies with repeated assessments have shown that financial and health literacy decline with advancing age and that these declines are associated with poorer decision making, reduced psychological well‐being, and other adverse functional outcomes.^7^, ^8^, ^9^ Moreover, literacy has been associated with incident Alzheimer's disease (AD) dementia and with neuropathological markers of neurodegeneration, suggesting that changes in literacy may reflect underlying brain aging processes.^10^, ^11^, ^12^, ^13^ Importantly, substantial heterogeneity exists in literacy trajectories across older adults, indicating that certain biological, behavioral, or environmental factors may influence the rate of decline.^7^, ^14^ Identifying such factors may help detect individuals at risk of functional vulnerability earlier in the aging process.

Sleep is a potential contributor to aging‐related changes in functioning and has received increasing attention in recent years. Sleep disturbances are common among older adults and have been linked to cognitive decline, AD, and related neurobiological processes. For example, sleep fragmentation has been associated with incident AD and faster cognitive decline in older adults,^15^ and sleep disturbances have been associated with altered cognitive trajectories in both preclinical and symptomatic stages of AD.^16^ Other studies have reported associations of both short and long sleep duration, as well as greater sleep variability, with worse cognitive performance or a higher risk of cognitive impairment in aging populations. However, many of these studies relied primarily on self‐reported sleep and focused on neuropsychological outcomes rather than functional abilities such as literacy.^17^, ^18^, ^19^, ^20^

Despite growing interest in sleep and cognitive aging, most studies have focused on neuropsychological performance or dementia risk rather than functional abilities that influence everyday decision making. Financial and health literacy represent complex higher order abilities that require integration of knowledge, attention, and judgment in real‐world contexts. These abilities may be particularly sensitive to changes in brain and behavioral health that accompany aging. In addition, prior research has often relied on self‐reported sleep measures, which may not fully capture sleep behavior in older adults. In particular, self‐reported measures may not adequately distinguish between nighttime sleep and daytime napping. Objective measures may therefore provide a more precise characterization of sleep behaviors relevant to functional outcomes.

The Rush Memory and Aging Project (MAP) provides a unique opportunity to examine these relationships. MAP is a community‐based cohort study of aging with annual clinical evaluations, repeated assessments of financial and health literacy, and objective sleep measurement using actigraphy.^21^ These features allow examination of both baseline literacy and longitudinal change in literacy over time.

In the present study, we examined associations between sleep characteristics and change in financial and health literacy in older adults without dementia. We evaluated both subjective and objective sleep measures, including self‐reported nighttime sleep duration, actigraphy‐derived nighttime sleep duration, and actigraphy‐derived daytime napping. We first examined associations between sleep and baseline literacy and then tested whether these sleep characteristics were associated with subsequent literacy decline using longitudinal models. We hypothesized that objective sleep measures, particularly nighttime sleep duration and daytime napping, would be associated with literacy level and change over time.

## 2 METHODS

### 2.1 Participants

We analyzed data from MAP,^21^ a prospective community‐based cohort study of aging and cognition conducted at the Rush Alzheimer's Disease Center. MAP began in 1997 and enrolls older adults from retirement communities, subsidized senior housing, and other community organizations in the Chicago metropolitan area. Participants undergo annual structured clinical evaluations. A sleep substudy beginning in 2005 collected wrist actigraphy and questionnaire‐based sleep measures. A decision‐making substudy introduced in 2010 included annual assessments of financial and health literacy.^22^, ^23^ Both MAP and the decision‐making substudy were approved by the institutional review board (Protocol Number 07071201) at Rush University Medical Center, and all participants provided written informed consent.

**RESEARCH IN CONTEXT**

1. **Systematic review**: Prior studies have linked sleep to cognitive decline and dementia risk, but few have examined functional outcomes such as financial and health literacy. Evidence on daytime napping and cognitive aging remains mixed, and longitudinal studies with objective sleep measures and repeated functional assessments are limited.
2. **Interpretation**: In this longitudinal cohort study of older adults, longer objectively measured nighttime sleep was associated with higher literacy levels. Greater daytime napping was associated with slower literacy decline. The nighttime sleep association with decline was observed in single‐predictor models but was attenuated and no longer statistically significant after mutual adjustment for napping. Self‐reported sleep duration was not associated with literacy trajectories in primary analyses, although short sleep (< 6 hours) was linked to lower baseline literacy. Findings were similar when financial and health literacy were examined separately. These correlational results extend prior work by linking sleep behavior to a functional outcome relevant to everyday decision making.
3. **Future directions**: Future studies should include repeated sleep assessments, more diverse samples, and biomarkers to clarify mechanisms and directionality. Better characterization of sleep timing and napping patterns may help separate compensatory from disease‐related processes. Intervention studies, and later follow‐up with literacy as an outcome, are needed to test whether changing sleep can help preserve functional abilities in older adults.

For these analyses, the analytic baseline was defined as the first visit at which participants completed the financial and health literacy assessment as part of the decision‐making substudy. Participants were required to be free of dementia at that visit, have valid literacy data, and contribute at least one subsequent literacy assessment. Dementia status was determined through a standardized clinical evaluation including medical history, neurological examination, and neuropsychological testing, followed by clinician classification using established criteria.^21^ Of the 2573 MAP participants, 2494 completed the initial study evaluation. Of these, 1401 had valid objective and subjective sleep measures. Among those with sleep data, 872 had valid financial and health literacy data and at least one subsequent literacy assessment. Of these participants, 833 were free of dementia at analytic baseline. Among these, 765 had valid objective and subjective sleep measures at the literacy baseline and comprised the analytic sample for the primary longitudinal analyses.

### 2.2 Assessment of financial and health literacy

Financial and health literacy was assessed at analytic baseline and annually thereafter using a validated 32‐item instrument developed for older adults.^10^, ^24^, ^25^, ^26^ The measure was designed to capture knowledge and use of common financial and health concepts relevant to everyday decision making in later life. The instrument consists of 32 items, including 23 financial literacy items and 9 health literacy items. Items cover topics such as compound interest, stocks and bonds, treatment‐associated risk, and common causes of mortality in older adults.^24^ Many financial literacy items were adapted from measures used in the Health and Retirement Study.^27^ Question formats include true/false and multiple‐choice responses.

Each item was scored as correct or incorrect, and percent correct was calculated separately for the financial literacy (23 items) and health literacy (9 items) components. The two percentages were then averaged to obtain a composite measure of total literacy (range: 0%–100%), with higher values indicating better literacy.^10^, ^24^ Financial and health literacy scores are moderately correlated (Pearson *r* ≈ 0.44) and the composite measure captures both domains while providing a well‐behaved distribution for statistical analyses.^10^

This literacy measure has demonstrated good internal consistency (Cronbach α ≈ 0.83) and has been associated with health‐promoting behaviors, functional status, hospitalization, and mortality in older adults.^24^, ^26^, ^28^, ^29^ In the present study, total literacy was analyzed as a continuous outcome in both cross‐sectional and longitudinal models.

### 2.3 Assessment of sleep measures

Sleep was assessed using both questionnaire‐based measures and wrist actigraphy.

#### 2.3.1 Subjective sleep measures

Subjective nighttime sleep duration was assessed using the MAP 32‐item sleep questionnaire, which includes selected items from the Pittsburgh Sleep Quality Index (PSQI), the Berlin Questionnaire, and the Mayo Sleep Questionnaire.^30^ The questionnaire was modified to reduce participant burden, and the present analyses did not use the modified global PSQI score. For this analysis, we used only the PSQI sleep duration item assessing habitual hours of sleep per night at the literacy baseline assessment. The questionnaire did not include a separate nap duration item, so self‐reported sleep duration could include some daytime sleep.

#### 2.3.2 Wrist actigraphy and derived sleep/rest–activity metrics

Objective sleep measures were derived from wrist actigraphy using previously published processing approaches.^31^, ^32^ We used two primary variables: nighttime sleep duration (sleep_duration_2100_700_avg) and daytime napping (nap_duration_in_min_per_day). Monitoring with the Actical device (Philips Respironics) was implemented in the MAP cohort beginning in 2005 and continued through 2018. Participants wore the device continuously on the non‐dominant wrist for a mean of ≈ 10 days (standard deviation [SD], ≈ 1 day). Napping in these analyses was quantified as the mean minutes of scored sleep per day across that wear period, rather than sleep on a single testing day. For each participant, the actigraphy recording used in these analyses was the recording aligned to the literacy analytic baseline (i.e., the sleep assessment at or closest to the first literacy visit), rather than a single calendar year for the entire cohort. The literacy evaluation and actigraphy wear were not required to occur on the same calendar day. Sleep questionnaires were collected in conjunction with the sleep assessment protocol. Although some participants contributed additional sleep assessments at other visits, the present study used a single baseline sleep assessment per participant. The Actical device is an omnidirectional accelerometer that samples at 32 Hz and integrates these signals into activity counts in 15‐second epochs.

Activity recordings underwent quality control using previously described preprocessing procedures. Periods of extreme activity counts and intervals consistent with non‐wear were identified and excluded based on established criteria. Gap metrics were calculated to characterize data completeness across recording days.

Sleep and wake states were estimated using a previously validated actigraphy‐based sleep scoring algorithm based on wrist activity counts.^33^, ^34^ To implement this algorithm, activity data were resampled into 1‐minute epochs. For each epoch, sleep or wake status was determined using a weighted activity score calculated from activity counts in the current epoch and surrounding epochs, specifically four preceding and two following epochs. Epochs with a weighted activity score < 1 were classified as sleep, and all other epochs were classified as wake.

Nighttime sleep duration was estimated using the same algorithm within a standardized nocturnal window from 9:00 pm to 7:00 am. Daytime napping was defined solely from actigraphy as sleep episodes occurring between 9:00 am and 7:00 pm, a common daylight or daily active‐hour window used for MAP Actical‐derived variables.^31^, ^32^ Those clock windows were chosen so the same night and day definitions could be applied to everyone and to reduce overlap with morning or evening transition periods. Sleep between 7:00 am and 9:00 am and between 7:00 pm and 9:00 pm is not assigned to either the nighttime or the nap variable. Nap detection used the same sleep/wake algorithm. Prior validation work supports applying this approach to daytime as well as nighttime sleep,^33^ although brief inactivity may still be misclassified as naps. If two nap segments occurred within 3 minutes of each other, they were merged and treated as a single nap episode. Mean daily nap duration was calculated as the average number of minutes spent napping per day across valid recording days.

Average nighttime sleep duration and average daytime nap duration were calculated across valid monitoring days for each participant. Because daytime nap duration was right‐skewed, values were square‐root transformed prior to statistical analyses. Twenty‐four‐hour activity acrophase was derived from the same actigraphy recording and represents the clock time of peak activity in the fitted 24‐hour rest–activity rhythm. Later values indicate later activity timing.

### 2.4 Cognitive assessment

Participants completed an annual neuropsychological battery comprising 19 tests assessing episodic memory, semantic memory, working memory, perceptual speed, and visuospatial ability. Raw scores were converted to *z* scores using the baseline mean and SD of the parent cohort and averaged to derive a composite measure of global cognition. Clinical diagnoses of dementia were determined through annual evaluations using established procedures in the MAP cohort.^35^, ^36^

### 2.5 Covariates

All participants self‐reported their sex (male or female), date of birth, and years of education. Age was calculated based on the time between the reported birth date and the date of baseline literacy assessment. Depressive symptoms were assessed with a 10‐item version of the Center for Epidemiologic Studies‐Depression Scale.^37^ For additional sensitivity analyses, diabetes was defined using a composite indicator based on hemoglobin A1c, self‐report, or diabetes medication use.^38^ Insomnia medication use was derived from medication data, and body mass index (BMI) was obtained from the clinical evaluation.^39^

### 2.6 Statistical analyses

Baseline characteristics of participants were summarized using means and SDs, medians and interquartile ranges (IQRs), or frequencies and percentages as appropriate. Included and non‐included MAP participants were also compared descriptively to characterize selection related to analytic eligibility. Pearson correlation coefficients were calculated to examine bivariate associations among baseline sleep measures, literacy scores, and global cognitive function.

Associations between sleep measures and financial and health literacy were examined using linear mixed‐effects models with annual literacy scores as the longitudinal continuous outcome. Separate models were estimated for self‐reported sleep duration, actigraphy‐derived nighttime sleep duration, and daytime nap duration. Time since baseline (years) was used as the time scale. Models included participant‐specific random intercepts and random slopes for time to account for individual differences in baseline literacy and rate of change over follow‐up.

The base model included terms for time, age, sex, and education, along with interactions between time and the demographic variables. Subsequent models were estimated sequentially. Model 1 additionally included the sleep exposure and the interaction between sleep and time. Model 2 further adjusted for baseline global cognition and the interaction between cognition and time. Model 3 additionally adjusted for depressive symptoms and their interaction with time.

Because daytime napping and nighttime sleep duration may capture related but distinct aspects of sleep behavior, additional models included both actigraphy‐derived nighttime sleep duration and daytime nap duration simultaneously, along with the same demographic, cognitive, and depressive symptom covariates.

Model assumptions were evaluated through inspection of residual plots, and multicollinearity among predictors was assessed using variance inflation factors.

We also conducted linear mixed‐effects models with annual global cognition as the longitudinal outcome, using the same model sequence as for literacy except that baseline cognition was not included as a covariate. In additional analyses, we repeated Model 3 separately for financial literacy and for health literacy.

Several sensitivity analyses were conducted to evaluate the robustness of the primary findings. First, sleep duration and daytime nap duration were categorized to examine potential non‐linear associations between sleep behavior and literacy trajectories. Sleep duration categories were defined based on commonly used thresholds in sleep research, distinguishing shorter, typical, and longer sleep durations. Self‐reported sleep duration was categorized into short (< 6 hours/night), normal (6–8 hours/night), and long (> 8 hours/night).^18^, ^20^ Because only eight participants (1.0%) had actigraphy‐derived nighttime sleep duration ≥ 8 hours, the normal and long categories were combined for objective nighttime sleep duration in the analysis. Napping time was categorized according to mean daily nap duration: < 30 minutes, 30 to < 60 minutes, 60 to < 120 minutes, and ≥ 120 minutes.^40^

Second, analyses were repeated after excluding participants who developed dementia within 2 years of the literacy baseline to reduce the potential influence of incident dementia on both sleep behavior and literacy change.

Third, analyses were repeated after excluding participants who developed dementia at any point during follow‐up to assess whether the observed associations were driven by individuals who later progressed to dementia.

Fourth, analyses were repeated after excluding extreme napping values (< 15 minutes/day or > 4 hours/day), following prior MAP actigraphy work.^32^ Minimal napping of < 15 minutes/day may not represent a meaningful nap habit, whereas > 4 hours/day may reflect highly fragmented daytime sleep. Primary models retained the full continuous napping distribution.

Fifth, to examine whether the fixed clock windows influenced the findings, Model 3 was repeated after adding centered 24‐hour activity acrophase and its interaction with time. Acrophase was also divided at the median into earlier and later groups to test whether the associations of nighttime sleep or napping with literacy change differed by activity timing.

Sixth, additional sensitivity models added diabetes, insomnia medication use, BMI, and their interactions with time to Model 3, first separately and then simultaneously.

All analyses were conducted using R software (version 4.5.1). Statistical tests were two sided with α = 0.05. Analyses were completed in August 2025.

## 3 RESULTS

### 3.1 Participant characteristics

The primary analysis included 765 participants who contributed 5419 annual literacy assessments across baseline and follow‐up visits. Participants had a mean age of 80.86 years (SD 7.48), 77.5% were women, and mean education was 15.41 years (SD 2.96). Baseline global cognition score averaged 0.19 (SD 0.51), and baseline literacy averaged 68.80% correct (SD 14.15). Self‐reported sleep duration averaged 7.23 hours/night (SD 1.27). Actigraphy‐derived nighttime sleep duration within the 9:00 pm to 7:00 am window averaged 5.18 hours (SD 1.38). Daytime napping was common, with a median duration of 54.4 minutes/day (IQR 27.4–93.9). At baseline, 80.0% were not cognitively impaired and 20.0% were classified as having mild cognitive impairment (MCI). During a median follow‐up of 7.0 years (IQR 3.9–10.0), 275 participants developed MCI and 252 developed dementia, contributing a median of 7 visits per participant (IQR 4–9). Baseline characteristics of the analytic sample are shown in Table 1. Participants included in the analytic sample were slightly younger (80.9 vs. 82.2 years), more often women (77.5% vs. 71.5%), slightly more educated (15.4 vs. 15.0 years), and had higher baseline cognition (0.19 vs. 0.05) than those not included. Baseline literacy was similar between the groups (68.8% vs. 68.4%; Table S1 in supporting information).

**TABLE 1:** Baseline participants characteristics.

Characteristic	Value
Age, years	80.86 (7.48)
Women, *n* (%)	593 (77.5)
Education, years	15.41 (2.96)
Cognitive normal, *n* (%)	612 (80.0%)
Mild cognitive impairment, *n* (%)	153 (20.0%)
Global cognition	0.19 (0.51)
Literacy, % correct	68.80 (14.15)
Depressive symptoms, median (IQR)	0.00 (0.00‐1.00)
Self‑reported sleep duration, h/night	7.23 (1.27)
Objective nighttime sleep, h/night	5.18 (1.38)
Daytime nap duration, min/day, median (IQR)	54.40 (27.40‐93.90)
Subjective sleep category: < 6 h	59 (7.7%)
Subjective sleep category: ≥ 8 h	339 (44.3%)
Objective nighttime sleep category: < 6 h	541 (70.7%)
Daytime napping category: 30–59 min/day	207 (27.1%)
Daytime napping category: 60–119 min/day	221 (28.9%)
Daytime napping category: ≥ 120 min/day	124 (16.2%)

Abbreviations: h, hours; IQR, interquartile range; min, minutes.

Correlations among baseline sleep measures, literacy, and global cognition are shown in Figure S1 in supporting information. Baseline literacy was moderately correlated with global cognition (*r* = 0.60). Correlations between sleep measures and literacy were modest, with greater nap duration associated with slightly lower literacy (*r* = −0.12). Objective nighttime sleep duration showed little cross‐sectional correlation with literacy (*r* = 0.01). Older age was correlated with lower literacy (*r* = −0.28) and longer nighttime sleep (*r* = 0.17). Because older adults tended to sleep longer and have lower literacy, these opposing age‐related associations largely masked the unadjusted correlation between sleep and literacy. After adjustment for age, sex, and education, longer nighttime sleep was associated with higher baseline literacy (Model 1 β *=* 0.776, *p* = 0.016). This pattern indicates suppression (negative confounding) by age (full coefficients are reported in Tables S2 and S3 in supporting information). Self‐reported and actigraphy‐derived sleep duration were weakly correlated (*r* = 0.19).

### 3.2 Longitudinal associations between sleep measures and literacy trajectories

In the demographic base model, literacy declined at an average rate of 1 percentage point per year (β for time = −0.958, standard error [SE] = 0.067, *p* < 0.001). Older age was associated with lower literacy at baseline (β *=* −0.499, *p* < 0.001) and faster decline over time (β *=* −0.068, *p* < 0.001). Higher education was associated with higher baseline literacy (β *=* 1.61, *p* < 0.001). Men had higher baseline literacy than women (β *=* 4.517, SE = 1.049, *p* < 0.001), but literacy decline did not differ by sex (sex × time β *=* 0.030, SE = 0.142, *p* = 0.833; Table S2).

Results of the longitudinal mixed‐effects models are presented in Table 2 (full Model 3 coefficients in Table S3). Estimated associations of the primary sleep measures with literacy decline in the fully adjusted models are summarized in Figure 1, and predicted literacy decline is illustrated in Figure 2.

**TABLE 2:** Longitudinal associations between sleep measures and literacy trajectories.

Predictor	Effect	Model 1 β (SE)	*p*	Model 2 β (SE)	*p*	Model 3 β (SE)	*p*
**Self‐reported sleep duration**	Baseline literacy	0.214 (0.343)	0.533	0.339 (0.282)	0.230	0.204 (0.284)	0.474
	Time × sleep	−0.019 (0.051)	0.702	−0.023 (0.050)	0.640	−0.040 (0.050)	0.431
**Objective nighttime sleep duration**	Baseline literacy	0.776 (0.322)	0.016	0.726 (0.264)	0.006	0.650 (0.264)	0.014
	Time × sleep	0.153 (0.045)	0.001	0.145 (0.044)	0.001	0.137 (0.044)	0.002
**Daytime nap duration (sqrt)**	Baseline literacy	−0.242 (0.130)	0.062	−0.102 (0.107)	0.340	−0.069 (0.107)	0.519
	Time × nap	0.068 (0.018)	<0.001	0.073 (0.017)	<0.001	0.075 (0.017)	<0.001

*Note*: Model 1 adds sleep predictors to the demographic base model. Model 2 additionally adjusts for baseline global cognition. Model 3 further adjusts for depressive symptoms.

Abbreviations: SE, standard error; sqrt, square root.

**FIGURE 1:**
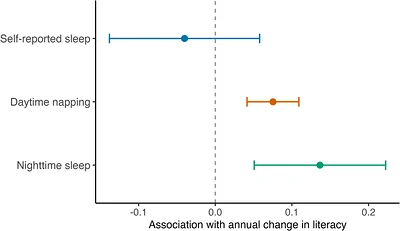
Associations of sleep characteristics with rate of change in literacy. Estimates from linear mixed‐effects models (Model 3) examining associations between sleep characteristics and annual change in literacy. Points represent regression estimates for the interaction between time and each sleep exposure, and horizontal lines indicate 95% confidence intervals. Positive estimates indicate slower decline in literacy over time. Models were adjusted for age, sex, and education, along with their interactions with time, and additionally included baseline global cognition and depressive symptoms, as well as their interactions with time. Each model included the sleep exposure and its interaction with time.

**FIGURE 2:**
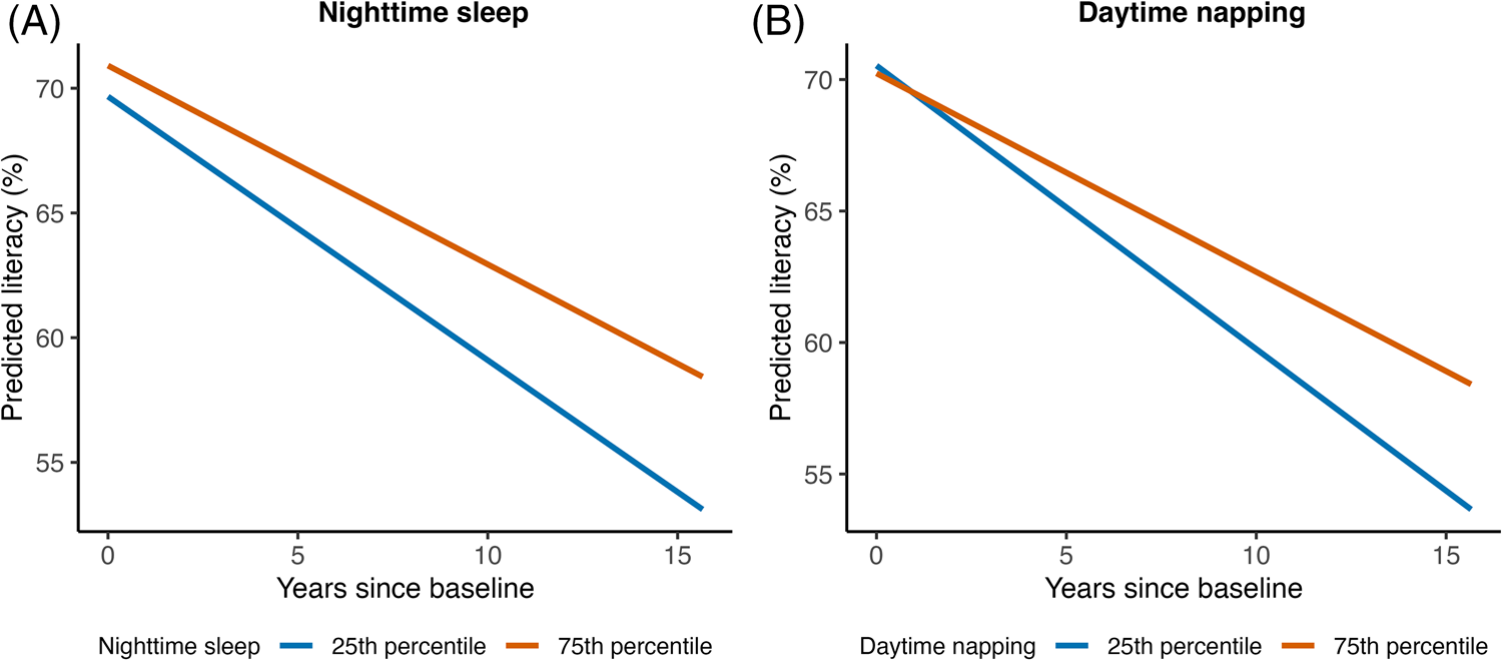
Predicted literacy trajectories by sleep characteristics. Predicted literacy trajectories from linear mixed‐effects models (Model 3) illustrating associations between sleep characteristics and change in literacy over time. Lines represent predicted literacy values for individuals at the 25th and 75th percentiles of each sleep measure, with other covariates held at their mean values or reference levels. Longer objective nighttime sleep duration was associated with higher baseline literacy and a slower rate of decline. Greater daytime napping was associated with a slower rate of literacy decline over time, despite minimal differences in baseline literacy.

Across models examining self‐reported sleep duration, there was no evidence that self‐reported sleep was associated with literacy level or rate of change over time. In the demographic‐adjusted model, each additional hour of self‐reported sleep was not associated with baseline literacy (β *=* 0.21, SE = 0.34, *p* = 0.53) or change in literacy over time (β *=* −0.02, SE = 0.05, *p* = 0.70). Results were similar after further adjustment for baseline cognition and depressive symptoms.

Longer actigraphy‐derived nighttime sleep duration was associated with both higher literacy level and slower literacy decline. In the demographic‐adjusted model (Model 1), each additional hour of nighttime sleep was associated with 0.78 percentage points higher literacy at baseline (β *=* 0.776, SE = 0.322, *p* = 0.016) and 0.15 percentage points per year slower decline (β *=* 0.153, SE = 0.045, *p* = 0.001). For context, the magnitude of the baseline association corresponds to ≈ 1.5 years of aging, and the effect on decline is roughly equivalent to offsetting ≈ 2 years of age‐related decline. These associations remained after adjustment for baseline cognition (baseline β *=* 0.726, *p* = 0.006; interaction β *=* 0.145, *p* = 0.001) and after further adjustment for depressive symptoms (baseline β *=* 0.650, *p* = 0.014; interaction β *=* 0.137, *p* = 0.002).

For daytime napping, the estimated association with baseline literacy was negative but did not reach statistical significance in the demographic‐adjusted model (β *=* −0.242, SE = 0.130, *p* = 0.062). In contrast, greater daytime napping was significantly associated with slower literacy decline over time. After further adjustment for baseline cognition and depressive symptoms, the estimate for baseline literacy was further attenuated, whereas the association with change over time remained significant (β *=* 0.075, SE = 0.017, *p* < 0.001).

To evaluate whether the associations of nighttime sleep and daytime napping were independent, both measures were included simultaneously in the same model (Model 4, full coefficients in Table S4 in supporting information). In this model, nighttime sleep remained associated with higher literacy levels (β *=* 0.882, SE = 0.293, *p* = 0.003), but its association with literacy decline was attenuated by approximately half and was no longer statistically significant (β *=* 0.074, SE = 0.048, *p* = 0.124). This attenuation suggests that part of the longitudinal association previously attributed to nighttime sleep is shared with daytime napping. Nighttime sleep and napping both capture 24‐hour rest–activity patterning, and the fixed clock windows may not align with the sleep schedules of people who sleep earlier or later. In contrast, greater nap duration remained associated with slower literacy decline (β *=* 0.063, SE = 0.019, *p* = 0.001), while its association with baseline literacy remained marginal. These combined model results are shown in Figure S2 in supporting information. Variance inflation factors (VIFs) were low (all VIF < 1.4), indicating no evidence of problematic multicollinearity.

### 3.3 Analyses with global cognition as the outcome

In linear mixed‐effects models with global cognition as the longitudinal outcome, longer daytime napping was associated with lower baseline cognition (β *=* −0.012, SE = 0.005, *p* = 0.018) but slower cognitive decline (nap × time β *=* 0.003, SE = 0.001, *p* = 0.008). Longer nighttime sleep was not associated with baseline cognition but was associated with slower cognitive decline (sleep × time β *=* 0.006, SE = 0.002, *p* = 0.016). Full coefficients are reported in Table S5 in supporting information.

### 3.4 Domain‐specific literacy analyses

When financial and health literacy were modeled separately, the pattern was similar across domains (Table S6 in supporting information). Baseline napping duration was not associated with either literacy domain. Longer daytime napping was associated with slower decline in both financial literacy (nap × time β *=* 0.052, SE = 0.019, *p* = 0.006) and health literacy (β *=* 0.091, SE = 0.022, *p* < 0.001). Longer nighttime sleep was associated with higher baseline financial literacy (β *=* 0.822, SE = 0.295, *p* = 0.005) and its association with financial literacy decline was similar in direction but did not reach statistical significance (β *=* 0.092, SE = 0.048, *p* = 0.055). The association of nighttime sleep with baseline health literacy was not significant (β *=* 0.505, SE = 0.351, *p* = 0.151), but longer nighttime sleep was associated with slower decline in health literacy (sleep × time β *=* 0.163, SE = 0.057, *p* = 0.005).

### 3.5 Sensitivity analyses

Results were robust across several sensitivity analyses. First, when sleep duration and nap duration were modeled categorically, the overall pattern of findings was similar (Table S7 in supporting information). Short self‐reported sleep (< 6 hours/night) was associated with lower baseline literacy (β *=* −4.01, *p* = 0.004) but was not associated with literacy decline. For objective sleep duration, sleeping < 6 hours/night was associated with lower baseline literacy (β *=* −1.48, *p* = 0.062) and faster decline (β *=* −0.360, *p* = 0.006). For daytime napping, compared to < 30 minutes/day, napping 60 to < 120 minutes/day and ≥ 120 minutes/day were associated with slower literacy decline (β *=* 0.449, *p* = 0.002 and β *=* 0.710, *p* < 0.001, respectively), while baseline literacy did not differ across nap categories. Predicted literacy trajectories are shown in Figure S3 in supporting information.

Second, after excluding participants who developed dementia within 2 years of the analytic baseline, the association between nap duration and literacy decline remained statistically significant but was slightly reduced in magnitude (Table S8 in supporting information). In this analysis, the nap × time estimate was 0.075 (SE 0.017, *p* < 0.001), while the baseline nap association remained non‐significant.

Third, when analyses were restricted to participants who remained dementia free throughout follow‐up, associations with literacy decline were attenuated for both nighttime sleep and daytime napping. Nighttime sleep × time decreased from β *=* 0.153 in Model 1 and β *=* 0.137 in Model 3 to β *=* 0.078 and β *=* 0.073, respectively. Nap × time decreased from β *=* 0.068 in Model 1 and β *=* 0.075 in Model 3 to β *=* 0.043 and β *=* 0.044, respectively, and all remained statistically significant. The proportional attenuation was similar for both exposures (Table S9 in supporting information).

Fourth, after excluding extreme nap values (< 15 minutes/day or > 4 hours/day), the association between napping and slower literacy decline remained statistically significant (β *=* 0.060, *p* = 0.018; Table S10 in supporting information).

Fifth, adjustment for 24‐hour activity acrophase did not materially change the Model 3 decline associations (Table S11 in supporting information). Tests of whether the associations of nighttime sleep or napping with literacy decline differed between later and earlier acrophase groups were not significant (*p* = 0.812 for nighttime sleep and *p* = 0.499 for napping).

Sixth, additional sensitivity analyses adjusting for diabetes, insomnia medication, and BMI yielded similar literacy findings (Table S12 in supporting information).

## 4 DISCUSSION

In this longitudinal study of older adults without dementia, objective sleep characteristics were associated with trajectories of financial and health literacy. Longer objectively measured nighttime sleep was associated with higher literacy levels and, in single‐predictor models, slower decline. The decline association was attenuated and no longer statistically significant after mutual adjustment for daytime napping. Greater daytime napping was associated with slower decline over time and was not significantly associated with baseline literacy. Self‐reported sleep duration was not associated with literacy level or change in the primary models, although short self‐reported sleep duration (< 6 hours) was associated with lower baseline literacy in categorical analyses. These findings suggest that sleep characteristics are associated with literacy trajectories in later life, but the associations are correlational rather than causal. Overall patterns were similar in models adjusting for cognition and depressive symptoms and across sensitivity analyses. To our knowledge, this is among the first longitudinal cohort studies examining objective sleep characteristics in relation to change in financial and health literacy.

Longer objectively measured nighttime sleep was consistently associated with higher baseline literacy across models. Its association with slower literacy decline was attenuated by approximately half and was no longer statistically significant after mutual adjustment for daytime napping (Model 4), indicating that the single‐predictor finding does not establish an association with decline independent of napping. This observation is broadly consistent with research linking disrupted sleep to adverse cognitive aging outcomes. Sleep fragmentation has been associated with incident AD and faster cognitive decline,^15^ and disrupted sleep has also been linked to neuroinflammatory processes and microglial aging accompanying cognitive impairment.^41^ More recent work has also linked sleep‐related biology and pericyte subtypes to cognitive decline in adults with and without AD.^42^ Sleep characteristics have also been associated with cognitive performance in preclinical and early symptomatic AD.^16^ Our findings extend this literature by focusing on literacy, which requires sustained attention, integration of new information with prior knowledge, and application of knowledge to complex real‐world decisions. Although actigraphy‐derived sleep duration estimates sleep from movement rather than directly measuring sleep architecture, it provides an objective measure of habitual sleep behavior and allows nighttime sleep and daytime napping to be measured separately.

For daytime napping, the estimated association with baseline literacy was generally negative but did not reach statistical significance in all models. In contrast, greater daytime napping was consistently associated with a slower rate of literacy decline across models and sensitivity analyses. This differs from prior MAP work showing that greater daytime napping was associated with increased risk of AD dementia.^40^ Differences in analytic framework and sample characteristics may contribute to these differing patterns. Prior studies used sleep assessment as baseline and examined incident dementia, whereas the present analyses were anchored at literacy assessment and examined longitudinal change in financial and health literacy. Measurement considerations may also contribute. Nighttime sleep and daytime napping were defined using fixed clock‐time windows, which may not capture individual differences in circadian timing. Individuals with later sleep schedules may have portions of their primary sleep period classified as daytime sleep, resulting in underestimation of nighttime sleep and overestimation of napping. We used the common MAP Actical windows so nighttime sleep and napping were defined the same way for everyone.^31^, ^32^, ^33^ Sleep after 7:00 am may be missed by the nighttime variable or, after 9:00 am, counted as a nap, while sleep occurring in the gaps between the predefined nighttime and daytime windows is assigned to neither variable. In sensitivity analyses adjusting for 24‐hour activity acrophase, the Model 3 sleep–literacy decline associations were largely unchanged (Table S11). Although activity acrophase cannot fully replace person‐specific sleep schedules, it provides a useful sensitivity check on rest–activity timing because it reflects peak activity rather than sleep onset or offset.

Daytime napping likely reflects multiple underlying mechanisms. Increased napping may indicate sleep fragmentation, daytime sleepiness, or emerging neurodegenerative changes, but may also represent a compensatory response to insufficient nighttime sleep or fatigue. Thus, the observed association between greater napping and slower literacy decline should be interpreted cautiously and may reflect compensatory behavior, preserved functional resilience, or measurement limitations rather than a direct protective effect. Experimental studies suggest that naps can support synaptic plasticity and learning‐related processes,^43^ while observational studies have reported heterogeneous associations of napping with brain and cognitive outcomes.^32^, ^44^, ^45^ Prior Rush work also reported a bidirectional relationship between daytime napping and AD dementia.^31^ Because the present study used a single baseline sleep assessment, it cannot capture how sleep behavior evolves in relation to literacy trajectories. Future studies with repeated sleep measurements may clarify whether changes in napping precede or follow changes in cognitive and functional outcomes. In analyses using global cognition as the longitudinal outcome (Table S5), greater daytime napping and longer nighttime sleep were associated with slower cognitive decline. Together, these results highlight the complexity of daytime sleep behavior and suggest that its implications may depend on nighttime sleep, nap timing, and changes in sleep patterns over time.

Another notable observation was the difference between objective and self‐reported sleep measures. Self‐reported sleep duration was not associated with literacy level or change in the primary models, although short sleep (< 6 hours) was associated with lower baseline literacy in categorical analyses. Self‐reported duration may combine actual sleep time with time spent in bed or fragmented sleep and may be influenced by recall or perceived sleep quality. In contrast, actigraphy provides a behaviorally specific characterization of sleep patterns and distinguishes nighttime sleep from daytime napping. Mean self‐reported nighttime sleep (7.23 hours) exceeded actigraphy‐derived nighttime sleep within the 9:00 pm to 07:00 am window (5.18 hours) by ≈ 2 hours (*r* = 0.19). Adding daytime nap duration (≈ 0.9 hours) did not reconcile this discrepancy, suggesting that the difference is not simply explained by nighttime sleep plus napping and is consistent with known differences between perceived sleep and movement‐based sleep estimation. Prior studies have also reported limited agreement between self‐reported and actigraphy‐derived sleep duration in older adults.^46^ In addition, the fixed nocturnal actigraphy window may underestimate nighttime sleep relative to self‐report when sleep occurs outside that window.

These findings have potential implications because financial and health literacy are important for effective decision making in later life. Lower literacy has been associated with poorer health, financial vulnerability, and increased mortality risk,^3^, ^4^, ^5^, ^6^ and literacy decline has been linked to poorer decision making and brain aging processes.^10^, ^47^ Because literacy integrates cognitive, motivational, and knowledge‐based processes that support real‐world decisions, identifying factors associated with literacy trajectories may help identify individuals at risk of functional vulnerability. Our findings suggest that sleep may represent one behavioral factor associated with literacy trajectories, but they do not establish that modifying sleep would alter these trajectories. Randomized sleep interventions with longer term follow‐up and literacy outcomes are needed to determine whether improving sleep can help preserve literacy or related functional abilities.

This study has several strengths, including a well‐characterized longitudinal cohort with repeated literacy assessments, objective actigraphy‐based characterization of nighttime sleep and daytime napping, and multiple sensitivity analyses. Literacy also represents a meaningful functional outcome closely linked to everyday decision making, extending the sleep literature beyond traditional cognitive outcomes. Several limitations should be considered. First, sleep and wake states were inferred using the Cole–Kripke algorithm, which was originally developed and validated using the Mini‐Motionlogger rather than the Actical device used here. Differences in signal processing may affect sleep classification, although prior studies have shown strong correlations in total sleep time between Actical and Motionlogger‐type devices when similar algorithms are applied.^48^ Participants did not provide sleep diaries or time‐in‐bed information, which may further affect accuracy. Second, participants were drawn from a volunteer cohort of predominantly White, relatively highly educated older adults recruited largely from retirement communities and community organizations in the Chicago metropolitan area, which may limit generalizability to racial and ethnic minority populations, individuals with lower socioeconomic status, or rural older adults. Studies with more diverse samples are ongoing. Third, the analytic sample comprised 765 of 2573 MAP participants with overlapping sleep and literacy assessments and complete covariate data. Included participants were slightly younger, more often women, slightly more educated, and had higher baseline cognition, with similar literacy. These differences are expected because the literacy baseline was later than the parent MAP baseline and dementia before literacy baseline was an exclusion criterion. Fourth, although analyses were longitudinal, the observational design limits causal inference, and bidirectional relationships cannot be excluded. Fifth, actigraphy cannot characterize sleep architecture or diagnose specific sleep disorders. Sensitivity models adjusting for diabetes, insomnia medication, and BMI yielded similar Model 3 associations, but Berlin apnea‐risk data were incomplete and psychiatric diagnoses beyond depressive symptoms were unavailable (Table S12). Future studies incorporating neuropathologic, cerebrovascular, and inflammatory biomarkers may help identify mechanisms linking sleep with literacy trajectories. Finally, detailed features of daytime napping, including timing and regularity, were not analyzed as primary exposures. Sleep exposures reflected ≈ 10 days of continuous actigraphy aligned with literacy baseline, so napping represented habitual daily sleep rather than a single nap immediately before testing. Literacy evaluation and actigraphy recording were matched at the visit level but were not required to occur on the same calendar day, and literacy testing time was not recorded. A same‐day nap is therefore unlikely to explain associations with literacy change over years, although an effect on baseline literacy cannot be ruled out. Activity acrophase provides a sensitivity check on rest–activity timing but does not define individualized sleep onset or offset. Additional analyses modeling financial and health literacy separately showed similar associations to the composite outcome (Table S6).

## 5 CONCLUSION

In conclusion, objective sleep characteristics were associated with financial and health literacy in older adults. Longer nighttime sleep was associated with higher literacy at baseline and, in single‐predictor models, slower decline over time, but the decline association was attenuated and no longer statistically significant after adjustment for napping. Greater daytime napping was associated with slower literacy decline, but not with baseline literacy. These correlational findings highlight literacy as a meaningful functional outcome related to brain health and aging. Further research with repeated sleep measurement and interventional designs is needed to clarify mechanisms and to determine whether modifying sleep can help preserve functional abilities in later life.

## CONFLICT OF INTEREST STATEMENT

The authors declare no conflicts of interest. Author disclosures are available in the Supporting Information.

## CONSENT STATEMENT

All participants provided written informed consent; individual studies were approved by an institutional review board at Rush University Medical Center.

## Supporting information



Supporting Information

Supporting Information

## Data Availability

Data used for this study can be requested for research purposes through the Rush Alzheimer's Disease Center Research Resource Sharing Hub (https://www.radc.rush.edu) subject to institutional approval and data‐use agreements.

## References

[1] Baker DW . The meaning and the measure of health literacy. J Gen Intern Med. 2006;21:878‐883. doi:10.1111/j.1525‐1497.2006.00540.x 10.1111/j.1525-1497.2006.00540.x PMC1831571 16881951

[2] Lusardi A , Mitchell OS . The economic importance of financial literacy: theory and evidence. J. Eco Lit. 2014;52:5‐44. doi:10.1257/jel.52.1.5 10.1257/jel.52.1.5 PMC5450829 28579637

[3] Dewalt DA , Berkman ND , Sheridan S , Lohr KN , Pignone MP . Literacy and health outcomes: a systematic review of the literature. J Gen Intern Med. 2004;19:1228‐1239. doi:10.1111/j.1525‐1497.2004.40153.x 10.1111/j.1525-1497.2004.40153.x PMC1492599 15610334

[4] Wolf MS , Gazmararian JA , Baker DW . Health literacy and functional health status among older adults. Arch Intern Med. 2005;165:1946‐1952. doi:10.1001/archinte.165.17.1946 10.1001/archinte.165.17.1946 16186463

[5] Lusardi A , Tufano P . Debt literacy, financial experiences, and overindebtedness. J Pension Econ Finance. 2015;14:332‐368.

[6] Baker DW , Wolf MS , Feinglass J , Thompson JA . Health literacy, cognitive abilities, and mortality among elderly persons. J Gen Intern Med. 2008;23:723‐726. doi:10.1007/s11606‐008‐0566‐4 10.1007/s11606-008-0566-4 PMC2517873 18330654

[7] Yu L , Mottola G , Bennett DA , Boyle PA . Adverse impacts of declining financial and health literacy in old age. Am J Geriatr Psychiatry. 2021;29:1129‐1139. doi:10.1016/j.jagp.2021.02.042 10.1016/j.jagp.2021.02.042 PMC8357843 33676832

[8] Finke MS , Howe JS , Huston SJ . Old age and the decline in financial literacy. Manag Sci. 2017;63:213‐230.

[9] Angrisani M , Burke J , Lusardi A , Mottola G . The evolution of financial literacy over time and its predictive power for financial outcomes: evidence from longitudinal data. J Pension Econ Finance. 2023;22:640‐657.

[10] Yu L , Wilson RS , Schneider JA , Bennett DA , Boyle PA . Financial and health literacy predict incident Alzheimer's disease dementia and pathology. J Alzheimers Dis. 2017;56:1485‐1493. doi:10.3233/JAD‐161132 10.3233/JAD-161132 PMC5444544 28157101

[11] Yu L , Schneider JA , Kapasi A , Bennett DA , Boyle PA . Limbic‐predominant age‐related TDP‐43 encephalopathy and distinct longitudinal profiles of domain‐specific literacy. Alzheimer Dis Assoc Disord. 2020;34:299‐305. doi:10.1097/WAD.0000000000000389 10.1097/WAD.0000000000000389 PMC7679283 32452861

[12] Kapasi A , Yu L , Stewart CC , Schneider JA , Bennett DA , Boyle PA . Association of TDP‐43 pathology with domain‐specific literacy in older persons. Alzheimer Dis Assoc Disord. 2019;33:315‐320. doi:10.1097/WAD.0000000000000334 10.1097/WAD.0000000000000334 PMC6878155 31305319

[13] Lamar M , Arfanakis K , Yu L , et al. The relationship of MRI‐Derived Alzheimer's and cerebrovascular‐related signatures with level of and change in health and financial literacy. Am J Geriatr Psychiatry. 2023;31:1129‐1139. doi:10.1016/j.jagp.2023.07.008 10.1016/j.jagp.2023.07.008 PMC10800641 37541932

[14] Kobayashi LC , Wardle J , Wolf MS . Cognitive Function and health literacy decline in a cohort of aging English adults. J Gen Intern Med. 2015;30:958‐964. doi:10.1007/s11606‐015‐3206‐9 10.1007/s11606-015-3206-9 PMC4471026 25680352

[15] Lim ASP , Kowgier M , Yu L , Buchman AS , Bennett DA . Sleep fragmentation and the risk of incident Alzheimer's disease and cognitive decline in older persons. Sleep. 2013;36:1027‐1032. doi:10.5665/sleep.2802 10.5665/sleep.2802 PMC3669060 23814339

[16] Lucey BP , Wisch J , Boerwinkle AH , et al. Sleep and longitudinal cognitive performance in preclinical and early symptomatic Alzheimer's disease. Brain. 2021;144:2852‐2862. doi:10.1093/brain/awab272 10.1093/brain/awab272 PMC8536939 34668959

[17] Keil SA , Schindler AG , Wang MX , et al. Longitudinal sleep patterns and cognitive impairment in older adults. JAMA Netw Open. 2023;6:e2346006. doi:10.1001/jamanetworkopen.2023.46006 10.1001/jamanetworkopen.2023.46006 PMC10696486 38048131

[18] Feng F , Cai Z , Chen J , Yang D , Huang N , Luo Y . U shaped association between sleep duration and long term cognitive decline trajectories in a national cohort. Sci Rep. 2025;15:39767. doi:10.1038/s41598‐025‐23440‐x 10.1038/s41598-025-23440-x PMC12615808 41233437

[19] Chen W , Wang X . Longitudinal associations between sleep duration and cognitive impairment in Chinese elderly. Front Aging Neurosci. 2022;14:1037650. doi:10.3389/fnagi.2022.1037650 10.3389/fnagi.2022.1037650 PMC9714471 36466606

[20] Lin L‐H , Xu W‐Q , Wang S‐B , et al. U‐shaped association between sleep duration and subjective cognitive complaints in Chinese elderly: a cross‐sectional study. BMC Psychiatry. 2022;22:147. doi:10.1186/s12888‐022‐03738‐0 10.1186/s12888-022-03738-0 PMC8867774 35209875

[21] Bennett DA , Buchman AS , Boyle PA , Barnes LL , Wilson RS , Schneider JA . Religious orders study and rush memory and aging project. J Alzheimers Dis. 2018;64:S161‐S189. doi:10.3233/JAD‐179939 10.3233/JAD-179939 PMC6380522 29865057

[22] Boyle PA , Yu L , Wilson RS , Gamble K , Buchman AS , Bennett DA . Poor decision making is a consequence of cognitive decline among older persons without Alzheimer's disease or mild cognitive impairment. PLoS One. 2012;7:e43647. doi:10.1371/journal.pone.0043647 10.1371/journal.pone.0043647 PMC3423371 22916287

[23] Boyle P , Yu L , Buchman A , Bennett D . Risk aversion is associated with decision making among community‐based older persons. Front Psychol. 2012;3:205. doi:10.3389/fpsyg.2012.00205 10.3389/fpsyg.2012.00205 PMC3385154 22754545

[24] Bennett JS , Boyle PA , James BD , Bennett DA . Correlates of health and financial literacy in older adults without dementia. BMC Geriatr. 2012;12:30. doi:10.1186/1471‐2318‐12‐30 10.1186/1471-2318-12-30 PMC3459814 22691341

[25] James BD , Boyle PA , Bennett JS , Bennett DA . The impact of health and financial literacy on decision making in community‐based older adults. Gerontology. 2012;58:531‐539. doi:10.1159/000339094 10.1159/000339094 PMC3491105 22739454

[26] Stewart CC , Yu L , Lamar M , Wilson RS , Bennett DA , Boyle PA . Associations of health and financial literacy with mortality in advanced age. Aging Clin Exp Res. 2020;32:951‐957. doi:10.1007/s40520‐019‐01259‐7 10.1007/s40520-019-01259-7 PMC6942237 31273677

[27] Mitchell OS , Lusardi A . Financial literacy and financial behavior at older ages. Wharton Pension Research Council Working Paper No. 2022‐01. January 09, 2022. doi:10.2139/ssrn.4006687

[28] Lamar M , Wilson RS , Yu L , et al. Associations of literacy with diabetes indicators in older adults. J Epidemiol Community Health. 2019;73:250‐255. doi:10.1136/jech‐2018‐210977 10.1136/jech-2018-210977 PMC6417101 30530520

[29] James BD , Wilson RS , Shah RC , et al. Association of financial literacy with hospitalization in community‐dwelling older adults. Med Care. 2018;56:596‐602. doi:10.1097/MLR.0000000000000932 10.1097/MLR.0000000000000932 PMC6004829 29901572

[30] Turner AD , Lim AS , Leurgans SE , Bennett DA , Buchman AS , Barnes LL . Self‐reported sleep in older African Americans and white Americans. Ethn Dis nd;26:521‐528. doi:10.18865/ed.26.4.521 10.18865/ed.26.4.521 PMC5072481 27773979

[31] Li P , Gao L , Yu L , et al. Daytime napping and Alzheimer's dementia: a potential bidirectional relationship. Alzheimers Dement. 2023;19:158‐168. doi:10.1002/alz.12636 10.1002/alz.12636 PMC9481741 35297533

[32] Gao C , Zheng X , Cai R , et al. Timing and intraindividual variability of daytime napping and Alzheimer's disease in older adults. Commun Med. 2025;5:219. doi:10.1038/s43856‐025‐00936‐1 10.1038/s43856-025-00936-1 PMC12145432 40483332

[33] Gao C , Li P , Morris CJ , et al. Actigraphy‐based sleep detection: validation with polysomnography and comparison of performance for nighttime and daytime sleep during simulated shift work. Nat Sci Sleep. 2022;14:1801‐1816. doi:10.2147/NSS.S373107 10.2147/NSS.S373107 PMC9581540 36275180

[34] Cole RJ , Kripke DF , Gruen W , Mullaney DJ , Gillin JC . Automatic sleep/wake identification from wrist activity. Sleep. 1992;15:461‐469. doi:10.1093/sleep/15.5.461 10.1093/sleep/15.5.461 1455130

[35] Bennett DA , Wilson RS , Schneider JA , et al. Natural history of mild cognitive impairment in older persons. Neurology. 2002;59:198‐205. doi:10.1212/wnl.59.2.198 10.1212/wnl.59.2.198 12136057

[36] Bennett DA , Schneider JA , Aggarwal NT , et al. Decision rules guiding the clinical diagnosis of Alzheimer's disease in two community‐based cohort studies compared to standard practice in a clinic‐based cohort study. Neuroepidemiology. 2006;27:169‐176. doi:10.1159/000096129 10.1159/000096129 17035694

[37] Wilson RS , Capuano AW , Boyle PA , et al. Clinical‐pathologic study of depressive symptoms and cognitive decline in old age. Neurology. 2014;83:702‐709. doi:10.1212/WNL.0000000000000715 10.1212/WNL.0000000000000715 PMC4150132 25080520

[38] Arvanitakis Z , Wilson RS , Bienias JL , Evans DA , Bennett DA . Diabetes mellitus and risk of Alzheimer disease and decline in cognitive function. Arch Neurol. 2004;61:661‐666. doi:10.1001/archneur.61.5.661 10.1001/archneur.61.5.661 15148141

[39] Arvanitakis Z , Capuano AW , Bennett DA , Barnes LL . Body mass index and decline in cognitive function in older black and white persons. J Gerontol A Biol Sci Med Sci. 2018;73:198‐203. doi:10.1093/gerona/glx152 10.1093/gerona/glx152 PMC5861969 28961897

[40] Leng Y , Redline S , Stone KL , Ancoli‐Israel S , Yaffe K . Objective napping, cognitive decline and risk of cognitive impairment in older men. Alzheimers Dement. 2019;15:1039‐1047. doi:10.1016/j.jalz.2019.04.009 10.1016/j.jalz.2019.04.009 PMC6699896 31227429

[41] Kaneshwaran K , Olah M , Tasaki S , et al. Sleep fragmentation, microglial aging, and cognitive impairment in adults with and without Alzheimer's dementia. Sci Adv. 2019;5:eaax7331. doi:10.1126/sciadv.aax7331 10.1126/sciadv.aax7331 PMC6905859 31844665

[42] Hamid M , Saha Detroja T , Tripathy SJ , et al. Sleep, pericyte subtypes and cognitive decline in adults with and without Alzheimer's disease. Brain. 2025;148:3379‐3391. doi:10.1093/brain/awaf161 10.1093/brain/awaf161 PMC12404711 40657931

[43] Fehér KD , Henckaerts P , Hirsch V , et al. A nap can recalibrate homeostatic and associative synaptic plasticity in the human cortex. NeuroImage. 2026;327:121723. doi:10.1016/j.neuroimage.2026.121723 10.1016/j.neuroimage.2026.121723 41544905

[44] Anderson EL , Richmond RC , Jones SE , et al. Is disrupted sleep a risk factor for Alzheimer's disease? Evidence from a two‐sample mendelian randomization analysis. Int J Epidemiol. 2021;50:817‐828. doi:10.1093/ije/dyaa183 10.1093/ije/dyaa183 PMC8271193 33150399

[45] Paz V , Dashti HS , Garfield V . Is there an association between daytime napping, cognitive function, and brain volume? A mendelian randomization study in the UK biobank. Sleep Health. 2023;9:786‐793. doi:10.1016/j.sleh.2023.05.002 10.1016/j.sleh.2023.05.002 PMC13344058 37344293

[46] Van Den Berg JF , Van Rooij FJ , Vos H , et al. Disagreement between subjective and actigraphic measures of sleep duration in a population‐based study of elderly persons. J Sleep Res. 2008;17:295‐302. doi:10.1111/j.1365‐2869.2008.00638.x 10.1111/j.1365-2869.2008.00638.x 18321246

[47] Stewart CC , Yu L , Kapasi A , Bennett DA , Boyle PA . APOE ε4 and decline in health and financial literacy in advanced age. J Am Geriatr Soc. 2025;74(4):1121‐1125. doi:10.1111/jgs.70291 10.1111/jgs.70291 PMC13080071 41472502

[48] Weiss AR , Johnson NL , Berger NA , Redline S . Validity of activity‐based devices to estimate sleep. J Clin Sleep Med. 2010;6:336‐342. PMC2919663 20726281

