# Supplementary material for: Objective sleep characteristics and longitudinal change in financial and health literacy in older adults
Source: Alzheimers Dement. 2026 Nov 1;22(10):e71948. doi: 10.1002/alz.71948 (PMC13724217; doi:10.1002/alz.71948)
Supplement: Supplementary file 2 [file ALZ-22-e71948-s001.docx]

**Supplementary Figure 1. Correlation matrix of baseline sleep measures, literacy, and global cognition**

Correlation matrix showing pairwise Pearson correlations among baseline sleep measures, literacy, and global cognition in the complete-case analytic sample. Sleep measures include self-reported nighttime sleep duration, actigraphy-derived nighttime sleep duration, and mean daily nap duration. Circle size and color intensity reflect the magnitude of correlations. Literacy was moderately correlated with global cognition, whereas correlations among sleep measures were modest.

**Supplementary Figure 2. Associations of nighttime sleep duration and daytime napping with rate of change in literacy in the combined model**

Estimates from linear mixed-effects models including both actigraphy-derived nighttime sleep duration and daytime napping simultaneously. Points represent regression estimates for the interaction between time and each sleep exposure, and horizontal lines indicate 95% confidence intervals. Positive estimates indicate slower decline in literacy over time. Models were adjusted for age, sex, education, baseline cognition, and depressive symptoms, along with their interactions with time.

**Supplementary Figure 3. Predicted literacy trajectories from categorical sleep models**

Predicted literacy trajectories from linear mixed-effects models using categorical sleep measures. Panel A shows self-reported nighttime sleep duration categories (<6 h, 6–<8 h, ≥8 h). Panel B shows actigraphy-derived nighttime sleep duration categories. Panel C shows daytime nap duration categories (<30 minutes, 30–<60 minutes, 60–<120 minutes, ≥120 minutes). Predictions are based on cognition-adjusted mixed-effects models with other covariates held at their mean values or reference levels.

**Supplementary Table 1 Included versus excluded MAP participants**

| Characteristic | Excluded | Included |
| --- | --- | --- |
| N | 1808 | 765 |
| Age, mean (SD) | 82.2 (7.5) | 80.9 (7.5) |
| Female, % | 71.5 | 77.5 |
| Education, mean (SD) | 15.0 (3.6) | 15.4 (3.0) |
| Baseline literacy, mean (SD) | 68.4 (15.4) | 68.8 (14.1) |
| Baseline cognition, mean (SD) | 0.05 (0.69) | 0.19 (0.51) |

Comparison of the analytic sample (included) with MAP participants not meeting analytic criteria (excluded). Literacy baseline is later than the parent MAP baseline.

**Supplementary Table 2 Base demographic model (full coefficients)**

| Predictor | Estimate (SE) | p |
| --- | --- | --- |
| (Intercept) | 69.705 (0.500) | <0.001 |
| Time (years) | −0.943 (0.066) | <0.001 |
| Age (centered) | −0.499 (0.058) | <0.001 |
| Male (vs female) | 4.515 (1.052) | <0.001 |
| Education (centered) | 1.613 (0.150) | <0.001 |
| Time × age | −0.068 (0.008) | <0.001 |
| Time × male | 0.031 (0.143) | 0.827 |
| Time × education | −0.019 (0.020) | 0.362 |

Demographic base model for literacy including time, age, sex, education, and demographic × time interactions (no sleep predictors). This is the base model described in the Results before sleep exposures are added. Analytic sample N = 765; 5,419 observations. Columns: Predictor, Estimate (SE), p.

**Supplementary Table 3 Model 3 fully adjusted single-predictor models (full coefficients for main-text Table 2)**

| Predictor | Estimate (SE) | p |
| --- | --- | --- |
| A. Nighttime sleep |  |  |
| (Intercept) | 66.960 (1.451) | <0.001 |
| Time (years) | −1.630 (0.238) | <0.001 |
| Age (centered) | −0.226 (0.051) | <0.001 |
| Male (vs female) | 6.244 (0.870) | <0.001 |
| Education (centered) | 0.769 (0.129) | <0.001 |
| Nighttime sleep (h) | 0.650 (0.264) | 0.014 |
| Baseline cognition (centered) | 14.655 (0.773) | <0.001 |
| Depressive symptoms (centered) | −0.700 (0.247) | 0.005 |
| Time × age | −0.063 (0.009) | <0.001 |
| Time × male | 0.114 (0.139) | 0.413 |
| Time × education | −0.043 (0.021) | 0.038 |
| Time × nighttime sleep | 0.137 (0.044) | 0.002 |
| Time × baseline cognition | 0.540 (0.131) | <0.001 |
| Time × depressive symptoms | −0.058 (0.045) | 0.194 |
| B. Daytime napping |  |  |
| (Intercept) | 70.875 (0.882) | <0.001 |
| Time (years) | −1.454 (0.139) | <0.001 |
| Age (centered) | −0.199 (0.051) | <0.001 |
| Male (vs female) | 5.887 (0.862) | <0.001 |
| Education (centered) | 0.768 (0.129) | <0.001 |
| Daytime napping (sqrt min) | −0.069 (0.107) | 0.519 |
| Baseline cognition (centered) | 14.607 (0.778) | <0.001 |
| Depressive symptoms (centered) | −0.748 (0.247) | 0.003 |
| Time × age | −0.065 (0.009) | <0.001 |
| Time × male | 0.040 (0.137) | 0.772 |
| Time × education | −0.048 (0.021) | 0.021 |
| Time × daytime napping | 0.075 (0.017) | <0.001 |
| Time × baseline cognition | 0.583 (0.130) | <0.001 |
| Time × depressive symptoms | −0.092 (0.044) | 0.038 |
| C. Self-reported sleep |  |  |
| (Intercept) | 68.954 (2.084) | <0.001 |
| Time (years) | −0.641 (0.365) | 0.080 |
| Age (centered) | −0.206 (0.050) | <0.001 |
| Male (vs female) | 5.826 (0.863) | <0.001 |
| Education (centered) | 0.770 (0.130) | <0.001 |
| Self-reported sleep (h) | 0.204 (0.284) | 0.474 |
| Baseline cognition (centered) | 14.662 (0.777) | <0.001 |
| Depressive symptoms (centered) | −0.729 (0.250) | 0.004 |
| Time × age | −0.058 (0.009) | <0.001 |
| Time × male | 0.062 (0.140) | 0.658 |
| Time × education | −0.048 (0.021) | 0.023 |
| Time × self-reported sleep | −0.040 (0.050) | 0.431 |
| Time × baseline cognition | 0.552 (0.132) | <0.001 |
| Time × depressive symptoms | −0.084 (0.045) | 0.064 |

Full fixed-effect coefficients for Model 3 corresponding to main-text Table 2 (demographics, baseline cognition, depressive symptoms, and time interactions). Panels: nighttime sleep, daytime napping, and self-reported sleep. Columns: Predictor, Estimate (SE), p.

**Supplementary Table 4 Model 4 joint nighttime sleep and daytime napping (full coefficients)**

| Predictor | Estimate (SE) | p |
| --- | --- | --- |
| (Intercept) | 67.372 (1.462) | <0.001 |
| Time (years) | −1.749 (0.239) | <0.001 |
| Age (centered) | −0.211 (0.051) | <0.001 |
| Male (vs female) | 6.430 (0.875) | <0.001 |
| Education (centered) | 0.772 (0.129) | <0.001 |
| Nighttime sleep (h) | 0.882 (0.293) | 0.003 |
| Daytime napping (sqrt min) | −0.227 (0.118) | 0.054 |
| Baseline cognition (centered) | 14.548 (0.774) | <0.001 |
| Depressive symptoms (centered) | −0.630 (0.249) | 0.012 |
| Time × age | −0.066 (0.009) | <0.001 |
| Time × male | 0.072 (0.138) | 0.604 |
| Time × education | −0.045 (0.020) | 0.028 |
| Time × nighttime sleep | 0.074 (0.048) | 0.124 |
| Time × daytime napping | 0.063 (0.019) | <0.001 |
| Time × baseline cognition | 0.571 (0.130) | <0.001 |
| Time × depressive symptoms | −0.078 (0.045) | 0.081 |

Nighttime sleep and daytime napping entered simultaneously with Model 3 covariates. Columns: Predictor, Estimate (SE), p.

**Supplementary Table 5 Analyses with global cognition as the outcome (full coefficients)**

| Predictor | Estimate (SE) | p |
| --- | --- | --- |
| A. Nighttime sleep |  |  |
| (Intercept) | 0.323 (0.070) | <0.001 |
| Time (years) | −0.101 (0.014) | <0.001 |
| Age (centered) | −0.023 (0.002) | <0.001 |
| Male (vs female) | −0.109 (0.042) | 0.010 |
| Education (centered) | 0.054 (0.006) | <0.001 |
| Nighttime sleep (h) | 0.001 (0.013) | 0.952 |
| Depressive symptoms (centered) | −0.041 (0.012) | <0.001 |
| Time × age | −0.005 (0.000) | <0.001 |
| Time × male | −0.004 (0.008) | 0.600 |
| Time × education | 0.001 (0.001) | 0.233 |
| Time × nighttime sleep | 0.006 (0.002) | 0.016 |
| Time × depressive symptoms | −0.005 (0.002) | 0.036 |
| B. Daytime napping |  |  |
| (Intercept) | 0.415 (0.042) | <0.001 |
| Time (years) | −0.089 (0.008) | <0.001 |
| Age (centered) | −0.022 (0.002) | <0.001 |
| Male (vs female) | −0.106 (0.041) | 0.010 |
| Education (centered) | 0.054 (0.006) | <0.001 |
| Daytime napping (sqrt min) | −0.012 (0.005) | 0.018 |
| Depressive symptoms (centered) | −0.038 (0.012) | 0.001 |
| Time × age | −0.005 (0.000) | <0.001 |
| Time × male | −0.008 (0.008) | 0.333 |
| Time × education | 0.001 (0.001) | 0.253 |
| Time × daytime napping | 0.003 (0.001) | 0.008 |
| Time × depressive symptoms | −0.006 (0.002) | 0.008 |
| C. Joint nighttime sleep and daytime napping |  |  |
| (Intercept) | 0.348 (0.070) | <0.001 |
| Time (years) | −0.105 (0.014) | <0.001 |
| Age (centered) | −0.022 (0.002) | <0.001 |
| Male (vs female) | −0.095 (0.042) | 0.024 |
| Education (centered) | 0.054 (0.006) | <0.001 |
| Nighttime sleep (h) | 0.017 (0.014) | 0.230 |
| Daytime napping (sqrt min) | −0.015 (0.006) | 0.008 |
| Depressive symptoms (centered) | −0.036 (0.012) | 0.003 |
| Time × age | −0.005 (0.000) | <0.001 |
| Time × male | −0.006 (0.008) | 0.492 |
| Time × education | 0.001 (0.001) | 0.238 |
| Time × nighttime sleep | 0.004 (0.003) | 0.152 |
| Time × daytime napping | 0.002 (0.001) | 0.071 |
| Time × depressive symptoms | −0.006 (0.002) | 0.018 |

Outcome is global cognition. Models adjust for demographics and depressive symptoms (and time interactions); baseline cognition is not a covariate. Columns: Predictor, Estimate (SE), p.

**Supplementary Table 6 Domain-specific financial and health literacy models (full coefficients)**

| Predictor | Estimate (SE) | p |
| --- | --- | --- |
| A. Financial literacy - nighttime sleep |  |  |
| (Intercept) | 70.418 (1.619) | <0.001 |
| Time (years) | −1.092 (0.257) | <0.001 |
| Age (centered) | −0.145 (0.057) | 0.010 |
| Male (vs female) | 12.285 (0.971) | <0.001 |
| Education (centered) | 0.712 (0.144) | <0.001 |
| Nighttime sleep (h) | 0.822 (0.295) | 0.005 |
| Baseline cognition (centered) | 13.434 (0.863) | <0.001 |
| Depressive symptoms (centered) | −0.692 (0.275) | 0.012 |
| Time × age | −0.044 (0.009) | <0.001 |
| Time × male | 0.048 (0.150) | 0.752 |
| Time × education | −0.044 (0.022) | 0.048 |
| Time × nighttime sleep | 0.092 (0.048) | 0.055 |
| Time × baseline cognition | 0.746 (0.141) | <0.001 |
| Time × depressive symptoms | −0.068 (0.048) | 0.160 |
| B. Financial literacy - daytime napping |  |  |
| (Intercept) | 74.544 (0.986) | <0.001 |
| Time (years) | −0.986 (0.150) | <0.001 |
| Age (centered) | −0.122 (0.057) | 0.034 |
| Male (vs female) | 11.811 (0.964) | <0.001 |
| Education (centered) | 0.709 (0.145) | <0.001 |
| Daytime napping (sqrt min) | 0.027 (0.119) | 0.821 |
| Baseline cognition (centered) | 13.430 (0.869) | <0.001 |
| Depressive symptoms (centered) | −0.778 (0.276) | 0.005 |
| Time × age | −0.045 (0.009) | <0.001 |
| Time × male | −0.001 (0.149) | 0.993 |
| Time × education | −0.048 (0.022) | 0.033 |
| Time × daytime napping | 0.052 (0.019) | 0.006 |
| Time × baseline cognition | 0.777 (0.141) | <0.001 |
| Time × depressive symptoms | −0.091 (0.048) | 0.057 |
| C. Health literacy - nighttime sleep |  |  |
| (Intercept) | 63.190 (1.925) | <0.001 |
| Time (years) | −1.937 (0.306) | <0.001 |
| Age (centered) | −0.313 (0.067) | <0.001 |
| Male (vs female) | 0.420 (1.151) | 0.716 |
| Education (centered) | 0.811 (0.171) | <0.001 |
| Nighttime sleep (h) | 0.505 (0.351) | 0.151 |
| Baseline cognition (centered) | 16.014 (1.026) | <0.001 |
| Depressive symptoms (centered) | −0.698 (0.328) | 0.034 |
| Time × age | −0.078 (0.011) | <0.001 |
| Time × male | 0.075 (0.176) | 0.672 |
| Time × education | −0.035 (0.027) | 0.192 |
| Time × nighttime sleep | 0.163 (0.057) | 0.005 |
| Time × baseline cognition | 0.229 (0.167) | 0.171 |
| Time × depressive symptoms | −0.055 (0.057) | 0.334 |
| D. Health literacy - daytime napping |  |  |
| (Intercept) | 66.923 (1.164) | <0.001 |
| Time (years) | −1.735 (0.177) | <0.001 |
| Age (centered) | −0.282 (0.068) | <0.001 |
| Male (vs female) | 0.145 (1.138) | 0.898 |
| Education (centered) | 0.816 (0.171) | <0.001 |
| Daytime napping (sqrt min) | −0.148 (0.141) | 0.294 |
| Baseline cognition (centered) | 15.923 (1.030) | <0.001 |
| Depressive symptoms (centered) | −0.709 (0.328) | 0.031 |
| Time × age | −0.081 (0.011) | <0.001 |
| Time × male | −0.007 (0.174) | 0.967 |
| Time × education | −0.042 (0.026) | 0.112 |
| Time × daytime napping | 0.091 (0.022) | <0.001 |
| Time × baseline cognition | 0.283 (0.168) | 0.093 |
| Time × depressive symptoms | −0.098 (0.057) | 0.084 |

Model 3 repeated separately for financial literacy and health literacy. Columns: Predictor, Estimate (SE), p.

**Supplementary Table 7 Categorical sleep duration and nap duration analyses**

| Predictor | Category | Baseline β (SE) | p | Time × predictor β (SE) | p |
| --- | --- | --- | --- | --- | --- |
| Self‑reported sleep | <6 h | ‑4.007 (1.392) | 0.004 | 0.246 (0.244) | 0.314 |
| Self‑reported sleep | ≥8 h | ‑0.364 (0.738) | 0.622 | 0.039 (0.120) | 0.743 |
| Objective sleep | <6 h | -1.475 (0.788) | 0.062 | -0.360 (0.131) | 0.006 |
| Nap duration | 30–<60 min | -0.175 (0.953) | 0.854 | 0.072 (0.143) | 0.614 |
| Nap duration | 60–<120 min | -0.992 (0.954) | 0.299 | 0.449 (0.147) | 0.002 |
| Nap duration | ≥120 min | -1.070 (1.138) | 0.347 | 0.710 (0.191) | <0.001 |

Categorical sensitivity analyses for self-reported sleep, objective nighttime sleep, and daytime napping.

**Supplementary Table 8 Sensitivity analysis excluding participants who developed dementia within 2 years of literacy baseline (full model coefficients)**

| Predictor | Estimate (SE) | p |
| --- | --- | --- |
| A. Nighttime sleep |  |  |
| (Intercept) | 66.887 (1.469) | <0.001 |
| Time (years) | −1.656 (0.237) | <0.001 |
| Age (centered) | −0.214 (0.051) | <0.001 |
| Male (vs female) | 6.099 (0.886) | <0.001 |
| Education (centered) | 0.813 (0.133) | <0.001 |
| Nighttime sleep (h) | 0.702 (0.268) | 0.009 |
| Baseline cognition (centered) | 13.845 (0.812) | <0.001 |
| Depressive symptoms (centered) | −0.635 (0.250) | 0.011 |
| Time × age | −0.062 (0.008) | <0.001 |
| Time × male | 0.083 (0.139) | 0.547 |
| Time × education | −0.037 (0.021) | 0.074 |
| Time × nighttime sleep | 0.148 (0.044) | <0.001 |
| Time × baseline cognition | 0.472 (0.132) | <0.001 |
| Time × depressive symptoms | −0.062 (0.045) | 0.161 |
| B. Daytime napping |  |  |
| (Intercept) | 70.838 (0.892) | <0.001 |
| Time (years) | −1.435 (0.138) | <0.001 |
| Age (centered) | −0.189 (0.052) | <0.001 |
| Male (vs female) | 5.703 (0.878) | <0.001 |
| Education (centered) | 0.807 (0.133) | <0.001 |
| Daytime napping (sqrt min) | −0.037 (0.108) | 0.736 |
| Baseline cognition (centered) | 13.847 (0.816) | <0.001 |
| Depressive symptoms (centered) | −0.695 (0.250) | 0.006 |
| Time × age | −0.064 (0.008) | <0.001 |
| Time × male | 0.008 (0.136) | 0.953 |
| Time × education | −0.041 (0.021) | 0.045 |
| Time × daytime napping | 0.077 (0.017) | <0.001 |
| Time × baseline cognition | 0.507 (0.132) | <0.001 |
| Time × depressive symptoms | −0.098 (0.044) | 0.026 |
| C. Self-reported sleep |  |  |
| (Intercept) | 67.792 (2.101) | <0.001 |
| Time (years) | −0.651 (0.364) | 0.074 |
| Age (centered) | −0.194 (0.051) | <0.001 |
| Male (vs female) | 5.620 (0.879) | <0.001 |
| Education (centered) | 0.813 (0.133) | <0.001 |
| Self-reported sleep (h) | 0.394 (0.288) | 0.171 |
| Baseline cognition (centered) | 13.869 (0.815) | <0.001 |
| Depressive symptoms (centered) | −0.643 (0.253) | 0.011 |
| Time × age | −0.058 (0.008) | <0.001 |
| Time × male | 0.029 (0.139) | 0.838 |
| Time × education | −0.043 (0.021) | 0.041 |
| Time × self-reported sleep | −0.034 (0.050) | 0.500 |
| Time × baseline cognition | 0.481 (0.134) | <0.001 |
| Time × depressive symptoms | −0.089 (0.045) | 0.048 |

Linear mixed-effects Model 3 restricted to participants who did not develop dementia within 2 years of the literacy baseline. Columns: Predictor, Estimate (SE), p.

**Supplementary Table 9 Sensitivity analysis excluding participants who developed dementia during follow-up (full model coefficients)**

| Predictor | Estimate (SE) | p |
| --- | --- | --- |
| A. Nighttime sleep |  |  |
| (Intercept) | 66.577 (1.636) | <0.001 |
| Time (years) | −0.966 (0.216) | <0.001 |
| Age (centered) | −0.190 (0.054) | <0.001 |
| Male (vs female) | 5.635 (0.967) | <0.001 |
| Education (centered) | 0.817 (0.145) | <0.001 |
| Nighttime sleep (h) | 0.787 (0.296) | 0.008 |
| Baseline cognition (centered) | 13.423 (0.920) | <0.001 |
| Depressive symptoms (centered) | −0.914 (0.269) | <0.001 |
| Time × age | −0.052 (0.008) | <0.001 |
| Time × male | 0.075 (0.121) | 0.534 |
| Time × education | −0.005 (0.019) | 0.785 |
| Time × nighttime sleep | 0.072 (0.040) | 0.070 |
| Time × baseline cognition | 0.025 (0.122) | 0.841 |
| Time × depressive symptoms | −0.024 (0.040) | 0.554 |
| B. Daytime napping |  |  |
| (Intercept) | 70.592 (0.995) | <0.001 |
| Time (years) | −0.908 (0.127) | <0.001 |
| Age (centered) | −0.166 (0.055) | 0.003 |
| Male (vs female) | 5.127 (0.956) | <0.001 |
| Education (centered) | 0.823 (0.146) | <0.001 |
| Daytime napping (sqrt min) | 0.023 (0.118) | 0.842 |
| Baseline cognition (centered) | 13.392 (0.927) | <0.001 |
| Depressive symptoms (centered) | −0.997 (0.269) | <0.001 |
| Time × age | −0.054 (0.008) | <0.001 |
| Time × male | 0.044 (0.120) | 0.715 |
| Time × education | −0.008 (0.019) | 0.658 |
| Time × daytime napping | 0.044 (0.016) | 0.005 |
| Time × baseline cognition | 0.043 (0.123) | 0.726 |
| Time × depressive symptoms | −0.047 (0.040) | 0.239 |
| C. Self-reported sleep |  |  |
| (Intercept) | 69.632 (2.262) | <0.001 |
| Time (years) | −0.246 (0.321) | 0.443 |
| Age (centered) | −0.163 (0.054) | 0.002 |
| Male (vs female) | 5.101 (0.956) | <0.001 |
| Education (centered) | 0.825 (0.146) | <0.001 |
| Self-reported sleep (h) | 0.164 (0.309) | 0.596 |
| Baseline cognition (centered) | 13.391 (0.926) | <0.001 |
| Depressive symptoms (centered) | −0.972 (0.272) | <0.001 |
| Time × age | −0.049 (0.007) | <0.001 |
| Time × male | 0.051 (0.121) | 0.671 |
| Time × education | −0.008 (0.019) | 0.668 |
| Time × self-reported sleep | −0.049 (0.045) | 0.276 |
| Time × baseline cognition | 0.029 (0.124) | 0.814 |
| Time × depressive symptoms | −0.043 (0.040) | 0.284 |

Linear mixed-effects Model 3 restricted to participants who remained dementia-free throughout follow-up. Columns: Predictor, Estimate (SE), p.

**Supplementary Table 10 Sensitivity analysis excluding extreme nap values (full model coefficients)**

| Predictor | Estimate (SE) | p |
| --- | --- | --- |
| (Intercept) | 70.061 (1.289) | <0.001 |
| Time (years) | −1.327 (0.208) | <0.001 |
| Age (centered) | −0.206 (0.056) | <0.001 |
| Male (vs female) | 6.163 (0.944) | <0.001 |
| Education (centered) | 0.903 (0.145) | <0.001 |
| Daytime napping (sqrt min) | 0.029 (0.151) | 0.846 |
| Baseline cognition (centered) | 14.192 (0.859) | <0.001 |
| Depressive symptoms (centered) | −0.795 (0.268) | 0.003 |
| Time × age | −0.061 (0.009) | <0.001 |
| Time × male | 0.089 (0.147) | 0.549 |
| Time × education | −0.049 (0.023) | 0.031 |
| Time × daytime napping | 0.063 (0.025) | 0.013 |
| Time × baseline cognition | 0.594 (0.142) | <0.001 |
| Time × depressive symptoms | −0.076 (0.047) | 0.109 |

Linear mixed-effects Model 3 for daytime napping after excluding extreme nap values (<15 minutes/day or >4 hours/day). Columns: Predictor, Estimate (SE), p.

**Supplementary Table 11 Acrophase sensitivity analyses (full coefficients)**

| Predictor | Estimate (SE) | p |
| --- | --- | --- |
| A. Nighttime sleep + acrophase |  |  |
| (Intercept) | 66.812 (1.488) | <0.001 |
| Time (years) | −1.638 (0.244) | <0.001 |
| Age (centered) | −0.218 (0.052) | <0.001 |
| Male (vs female) | 6.256 (0.877) | <0.001 |
| Education (centered) | 0.772 (0.130) | <0.001 |
| Nighttime sleep (h) | 0.681 (0.272) | 0.012 |
| Acrophase (centered) | 0.188 (0.253) | 0.457 |
| Baseline cognition (centered) | 14.778 (0.782) | <0.001 |
| Depressive symptoms (centered) | −0.737 (0.253) | 0.004 |
| Time × age | −0.063 (0.009) | <0.001 |
| Time × male | 0.106 (0.140) | 0.446 |
| Time × education | −0.041 (0.021) | 0.050 |
| Time × nighttime sleep | 0.140 (0.045) | 0.002 |
| Time × acrophase | −0.014 (0.041) | 0.737 |
| Time × baseline cognition | 0.535 (0.132) | <0.001 |
| Time × depressive symptoms | −0.053 (0.046) | 0.248 |
| B. Daytime napping + acrophase |  |  |
| (Intercept) | 70.811 (0.893) | <0.001 |
| Time (years) | −1.430 (0.139) | <0.001 |
| Age (centered) | −0.193 (0.052) | <0.001 |
| Male (vs female) | 5.876 (0.868) | <0.001 |
| Education (centered) | 0.768 (0.131) | <0.001 |
| Daytime napping (sqrt min) | −0.058 (0.108) | 0.593 |
| Acrophase (centered) | 0.094 (0.251) | 0.708 |
| Baseline cognition (centered) | 14.728 (0.787) | <0.001 |
| Depressive symptoms (centered) | −0.788 (0.253) | 0.002 |
| Time × age | −0.066 (0.009) | <0.001 |
| Time × male | 0.031 (0.137) | 0.823 |
| Time × education | −0.046 (0.021) | 0.027 |
| Time × daytime napping | 0.073 (0.017) | <0.001 |
| Time × acrophase | −0.035 (0.040) | 0.377 |
| Time × baseline cognition | 0.572 (0.131) | <0.001 |
| Time × depressive symptoms | −0.085 (0.045) | 0.060 |

Model 3 plus actigraphy-derived 24-h activity acrophase (centered) and time × acrophase. Columns: Predictor, Estimate (SE), p.

**Supplementary Table 12 Comorbidity-adjusted sensitivity analyses (full coefficients)**

| Predictor | Estimate (SE) | p |
| --- | --- | --- |
| A. Nighttime sleep |  |  |
| (Intercept) | 66.726 (1.487) | <0.001 |
| Time (years) | −1.657 (0.244) | <0.001 |
| Age (centered) | −0.229 (0.053) | <0.001 |
| Male (vs female) | 6.115 (0.885) | <0.001 |
| Education (centered) | 0.761 (0.133) | <0.001 |
| Nighttime sleep (h) | 0.665 (0.269) | 0.014 |
| Baseline cognition (centered) | 14.725 (0.783) | <0.001 |
| Depressive symptoms (centered) | −0.669 (0.252) | 0.008 |
| Diabetes (ser/sr/rx) | 0.767 (1.049) | 0.465 |
| Insomnia medication | 0.967 (1.228) | 0.431 |
| BMI (centered) | −0.057 (0.071) | 0.425 |
| Time × age | −0.060 (0.009) | <0.001 |
| Time × male | 0.135 (0.141) | 0.340 |
| Time × education | −0.039 (0.021) | 0.065 |
| Time × nighttime sleep | 0.136 (0.045) | 0.003 |
| Time × baseline cognition | 0.553 (0.132) | <0.001 |
| Time × depressive symptoms | −0.077 (0.046) | 0.089 |
| Time × diabetes (ser/sr/rx) | −0.129 (0.175) | 0.461 |
| Time × insomnia medication | 0.290 (0.190) | 0.129 |
| Time × BMI | 0.024 (0.012) | 0.038 |
| B. Daytime napping |  |  |
| (Intercept) | 70.593 (0.931) | <0.001 |
| Time (years) | −1.463 (0.146) | <0.001 |
| Age (centered) | −0.202 (0.054) | <0.001 |
| Male (vs female) | 5.762 (0.878) | <0.001 |
| Education (centered) | 0.761 (0.133) | <0.001 |
| Daytime napping (sqrt min) | −0.053 (0.113) | 0.640 |
| Baseline cognition (centered) | 14.707 (0.788) | <0.001 |
| Depressive symptoms (centered) | −0.726 (0.252) | 0.004 |
| Diabetes (ser/sr/rx) | 0.752 (1.055) | 0.476 |
| Insomnia medication | 1.109 (1.231) | 0.368 |
| BMI (centered) | −0.048 (0.073) | 0.512 |
| Time × age | −0.063 (0.009) | <0.001 |
| Time × male | 0.062 (0.140) | 0.655 |
| Time × education | −0.045 (0.021) | 0.034 |
| Time × daytime napping | 0.074 (0.018) | <0.001 |
| Time × baseline cognition | 0.591 (0.132) | <0.001 |
| Time × depressive symptoms | −0.104 (0.045) | 0.022 |
| Time × diabetes (ser/sr/rx) | −0.197 (0.175) | 0.261 |
| Time × insomnia medication | 0.326 (0.190) | 0.087 |
| Time × BMI | 0.016 (0.012) | 0.178 |

Model 3 plus diabetes (diabetes_ser_sr_rx), insomnia medication (insomnia_rx), and BMI, with time interactions. Columns: Predictor, Estimate (SE), p.
